# Insight into the Role of the BCL6B Gene in Biological Functions and Disease Progression

**DOI:** 10.7150/jca.116659

**Published:** 2025-08-16

**Authors:** Yuqing Pan, Ya Li

**Affiliations:** 1 Department of Clinical Laboratory, the First Affiliated Hospital of Kunming Medical University, Kunming, Yunnan, China.; 2 Yunnan Key Laboratory of Laboratory Medicine, Kunming, Yunnan, China.; 3 Yunnan Innovation Team of Clinical Laboratory and Diagnosis, the First Affiliated Hospital of Kunming Medical University, Kunming, Yunnan, China.; 4 Yunnan Province Clinical Research Center for Laboratory Medicine, Kunming, Yunnan, China.; 5 Department of Laboratory Medicine, the Affiliated Hospital of Yunnan University, Kunming, Yunnan, China.

**Keywords:** BCL6B, Tumor suppression, Immune regulation, Epigenetics, Angiogenesis, Stem cell homeostasis

## Abstract

The B-cell CLL/lymphoma 6B (BCL6B) gene, a homolog of *BCL6*, belongs to the ZBTB (zinc finger and BTB domain-containing) protein family and functions as a transcriptional repressor involved in gene regulation and cellular proliferation. In recent years, BCL6B has garnered increasing attention due to its critical involvement in various biological processes, including tumor suppression, immune modulation, stem cell maintenance, and angiogenesis. Moreover, its dysregulation, often through epigenetic modifications such as promoter hypermethylation, has been implicated in the pathogenesis of several malignancies and immune-related disorders. This review provides a comprehensive overview of BCL6B's molecular functions, its roles in human disease, and emerging research advances, highlighting its potential as both a diagnostic biomarker and a therapeutic target.

## Introduction

The BCL6B gene, also referred to as ZBTB28, BAZF,* or* ZNF62, belongs to the ZBTB transcription factor superfamily and is located approximately 800 kb upstream of the well-characterized tumor suppressor gene P53 1. Functioning primarily as a transcriptional repressor, BCL6B binds to specific DNA sequences to modulate gene expression, either repressing or indirectly promoting transcription depending on cellular context and cofactor interactions. Accumulating evidence suggests that BCL6B is a multifunctional transcription factor involved in diverse biological processes, including stem cell self-renewal, differentiation, proliferation, apoptosis, migration, invasion, angiogenesis, tumor growth in xenograft models, metastasis, cell cycle arrest, epithelial-mesenchymal transition (EMT) reversal, macrophage activation, reactive oxygen species (ROS) generation, and modulation of sensitivity to anticancer agents. BCL6B has been shown to play a critical role in the initiation and progression of multiple solid tumors, including hepatocellular carcinoma, gastric cancer, colorectal cancer, breast cancer, cervical cancer, and various lymphomas. Interestingly, while BCL6B expression is upregulated in differentiated thyroid carcinoma, it is frequently downregulated in many other solid tumors, primarily due to promoter hypermethylation. These findings highlight the potential of BCL6B as both a prognostic biomarker and a therapeutic target, while also offering mechanistic insights into its biological functions. This review provides an overview of BCL6B's structural features, biological activities, and the mechanisms by which it contributes to disease onset and progression, aiming to identify novel therapeutic strategies.

## BCL6B Gene Overview

### Historical Context and Discovery of BCL6B Gene

In 1993, the Department of Pathology at the University of Chicago identified B-cell lymphoma 6 (BCL6), the most frequently translocated or mutated oncogene on chromosome 3 in B-cell non-Hodgkin lymphoma 2^.^ In the same year, Ye BH et al. 3 proposed that BCL6 might be a candidate proto-oncogene in the pathogenesis of B-cell non-Hodgkin lymphoma. Research on BCL6B began with studies of the zinc finger motifs in BCL6, leading to its successful cloning in mice using zinc finger domain probes 4. As a proto-oncogene, BCL6 was first identified in lymphoma research, where it was shown to regulate B-cell proliferation and survival. Evidence indicates that BCL6 is overexpressed in multiple human malignancies, especially those involving the lymphatic system 5. Continued investigation into BCL6 resulted in the identification of its homologous gene, BCL6B.In 1998, Okabe S et al. 6 successfully characterized BCL6B, noting a 94% amino acid sequence similarity with BCL6 and analogous biochemical traits. Consequently, BCL6B interacts with BCL6 through the BTB/POZ domain, localizes in the nucleus, and binds BCL6-specific DNA sequences to act as a transcriptional repressor. Structurally, BCL6B harbors five zinc finger motifs, slightly fewer than BCL6's six, highlighting both its resemblance and distinct identity as a BCL6 variant. Subsequent studies have demonstrated that BCL6B plays pivotal roles in a wide range of biological activities. Moreover, BCL6B exhibits a unique tissue-specific expression profile, distinct from that of BCL6, suggesting it exerts context-specific and independent functions 7.* BCL6B*, localized in the nucleus, directly regulates gene transcription and plays a significant role in cell growth and differentiation.

### Chromosomal Location and Genomic Features of BCL6B

The BCL6B gene is situated on chromosome 17 (17p13.1) in humans, flanked by the Arachidonate 12-Lipoxygenase (ALOX12) and Asialoglycoprotein Receptor 2 (ASGR2) genes. The gene comprises 9 exons and 8 introns, forming a complex genomic structure (NCBI Gene ID: 255877). Alternative splicing of the BCL6B coding region produces six mRNA variants and multiple protein isoforms, each potentially contributing to unique cellular roles. BCL6B mRNA is widely expressed across human tissues, with the highest abundance observed in the heart and placenta. It is also found in various cell types, particularly in activated lymphocytes and certain tumor cells 3. In mouse models, predominant expression of BCL6B mRNA is observed in the heart and lung tissues 6.

### Structural Characteristics and Functional Domains of BCL6B Protein

**Figure 1:**
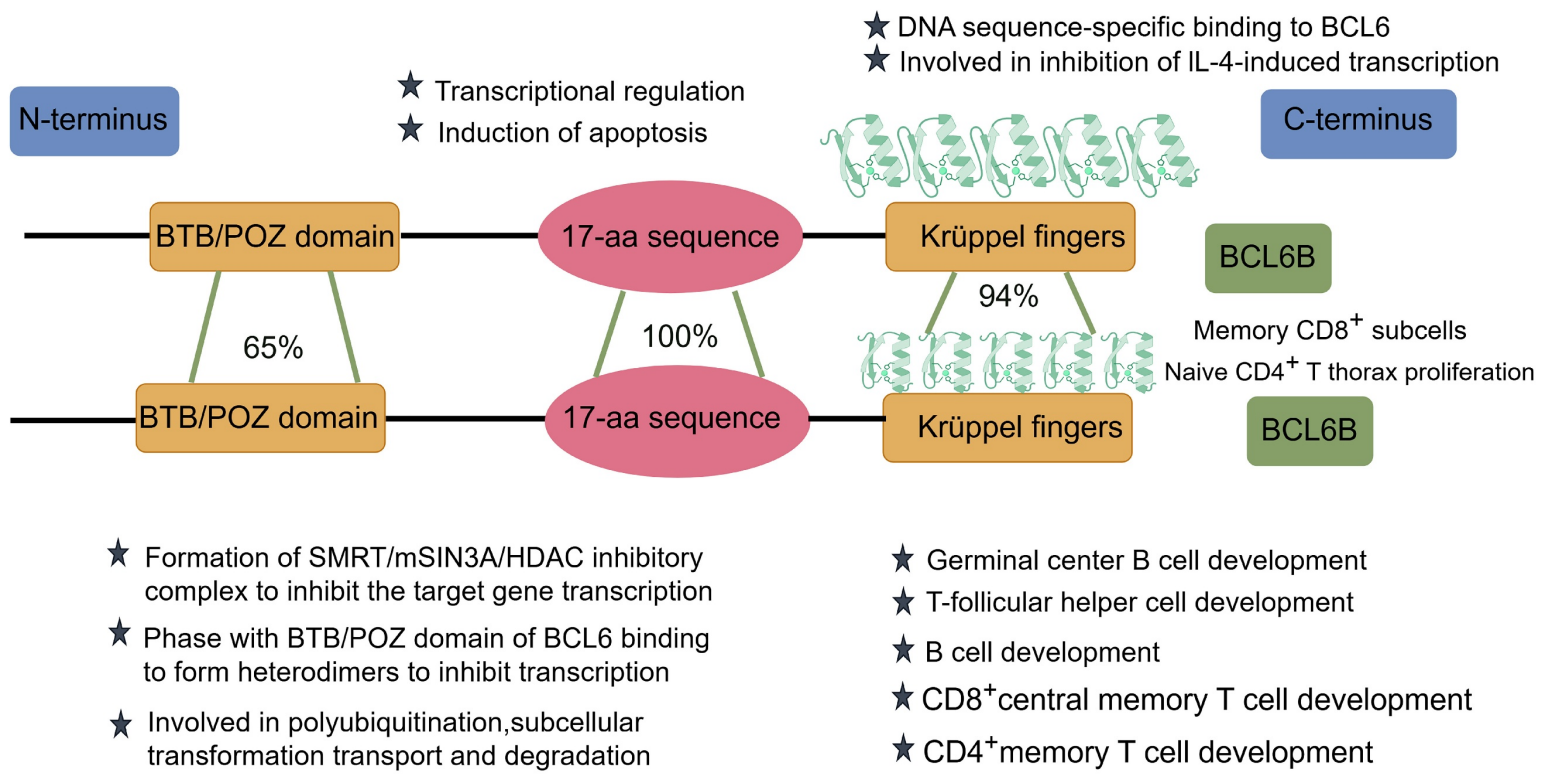
The structural diagram, similarity, and functions of BCL6 and BCL6B.

The BCL6B protein functions as a transcriptional repressor, utilizing zinc finger domains to bind specific DNA sequences and regulate target gene expression 8. It plays a regulatory role in key biological pathways, including cell cycle progression, apoptotic signaling, and DNA damage repair mechanisms. Comparative analyses indicate that BCL6B and BCL6 share three key structural domains essential for their transcriptional regulatory functions (Figure 1):

#### BTB/POZ Domain

At the N-terminus of BCL6B lies the BTB (Broad-complex, Tramtrack, and Bric-à-brac)/POZ (Pox virus and Zinc finger) domain, which shares approximately 65% sequence similarity with that of BCL6. This domain interacts with the mammalian Switch-Independent 3A (mSIN3A), the silencing mediator for retinoid and thyroid hormone receptors (SMRT), and histone deacetylase 1 (HDAC1), collectively forming the SMRT/mSIN3A/HDAC transcriptional repressor complex those silences downstream gene expression. Notably, BCL6B and BCL6 competitively regulate the expression of the tumor suppressor gene P53, with BCL6 itself being a direct target of ZBTB28. This dynamic interaction constitutes the BCL6B-BCL6-P53 regulatory axis, which plays a pivotal role in tumor suppression 9. Moreover, the BTB domain within ZBTB family members facilitates the formation of homo- or heterodimers, which are essential for protein stability, nuclear localization, and transcriptional control 10. Importantly, this domain also functions as an adaptor in Cullin 3 (CUL3)-based E3 ubiquitin ligase complexes, mediating the interaction with C-promoter binding factor 1 (CBF1) and promoting its polyubiquitination, transport, and subsequent proteasomal degradation 11. Additionally, the BTB domain is also thought to influence transcription by inducing nucleosome remodeling and changes in local chromatin structure 12.

#### Intermediate Region

The central region of *BCL6B* contains a conserved 17-amino-acid sequence that is 100% identical to the corresponding region in BCL6.This motif is believed to recruit histone deacetylases (HDACs), acting as a molecular bridge between the SMRT corepressor complex and the BTB/POZ domain. Through this interaction, BCL6B modulates the activity of retrotranscription factors, contributing substantially to transcriptional repression 6. In 2001, Hong ZH et al. 8 identified the 17-amino-acid region as another repressive domain shared by BCL6 and BCL6*B*, closely linked to apoptosis induction.

#### Zinc Finger Domain

At the C-terminus, BCL6B contains five tandemly repeated Cys2-His2-type Krüppel-like zinc finger domains, in contrast to the six present in BCL6, with a high overall sequence homology of 94%. Theα-helical region plays a critical role in determining DNA-binding sequence specificity. These zinc finger domains specifically bind to BCL6 DNA-binding sequences, which are similar to those of STAT, and may inhibit IL-4-induced transcription 6. The evolutionary conservation of these zinc finger domains suggests that BCL6B's functions have remained preserved throughout evolution. BCL6B is critical for diverse biological processes, such as tumor suppression, immune regulation, stem cell self-renewal, and angiogenesis 1.

## The Biological Functions of BCL6B

### Role of BCL6B in Transcriptional Repression

BCL6B exerts its biological functions—such as tumor suppression, immune modulation, stem cell maintenance, and angiogenesis—primarily through its role as a transcriptional repressor. Mechanistic studies have shown that BCL6B, often in cooperation with BCL6, binds to DNA sequences recognized by the signal transducer and activator of transcription (STAT) family, thereby inhibiting the transcription of STAT-responsive genes 13. Notably, BCL6B lacks the intrinsic capacity to independently recruit transcriptional repressor complexes. Instead, it requires heterodimerization via its BTB/POZ domain or its central 17-amino-acid conserved region to recruit the mSin3A/HDAC1 complex, mediating effective transcriptional repression 14.

In addition, *BCL6B* interacts with Cullin 3 (CUL3) and C-promoter binding factor 1 (CBF1), facilitating the ubiquitination and proteasomal degradation of CBF1, thus downregulating the Notch signaling pathway 10. These interactions underscore BCL6B's role as a key regulatory hub modulating transcriptional silencing in multiple biological contexts.

### Pathways Involving BCL6B

BCL6B is involved in the regulation of several key signaling pathways, such as Notch, VEGFR, STAT, PI3K/AKT, and p53, all of which play central roles in controlling cellular proliferation, differentiation, survival, and apoptosis 6, 11. Through modulation of these pathways, BCL6B influences tumor growth, metastasis, and resistance to therapy. Its involvement in these cascades offers new perspectives in understanding tumor biology and developing targeted treatments.

Future research should aim to delineate additional signaling networks influenced by BCL6B and clarify its context-specific mechanisms across various cancer types. These insights may uncover novel therapeutic targets and contribute to precision oncology.

### Cellular Functions of BCL6B

Aberrant BCL6B expression is associated with dysregulation of key cellular processes, including the inhibiting cell proliferation, inducing cell cycle arrest, promoting apoptosis, and downregulating anti-apoptotic proteins. These findings demonstrate that BCL6B exerts a dual regulatory role in cancer biology by simultaneously blocking tumor cell growth and facilitating programmed cell death.

Given its critical influence on both normal and malignant cell behavior, BCL6B is increasingly recognized as a pivotal regulator in tumor initiation and progression. Investigating its molecular mechanisms in greater depth may lead to innovative therapeutic approaches that leverage BCL6B's pro-apoptotic and anti-proliferative functions to combat cancer more effectively.

## Research Progress on BCL6B in Tumors

BCL6B is involved in a wide range of biological processes, including stem cell maintenance, angiogenesis, and immune regulation. Recent studies have increasingly focused on its potential roles in tumor initiation, progression, and prognosis. Tumorigenesis is a complex multistep process driven by the activation of oncogenes and inactivation of tumor suppressor genes, leading to dysregulated cell growth and eventual malignant transformation. As a transcriptional repressor, BCL6B regulates gene expression by modulating both oncogenic and tumor suppressive pathways, highlighting its potential as a biomarker and therapeutic target. Understanding the molecular mechanisms through which BCL6B affects tumor biology is therefore of significant clinical relevance 9.

Gene expression profiling indicates that BCL6B is broadly expressed in normal human tissues, with particularly high levels in adipose tissue, lung, and placenta. However, its expression patterns vary across tumor types. Data from The Cancer Genome Atlas (TCGA) reveal upregulated BCL6B expression in head and neck squamous cell carcinoma, cholangiocarcinoma, clear cell renal carcinoma, hepatocellular carcinoma, gastric cancer, thyroid cancer, and glioblastoma. Conversely, downregulated BCL6B expression is observed in bladder urothelial carcinoma, invasive breast cancer, chromophobe renal carcinoma, renal papillary cell carcinoma, lung adenocarcinoma, lung squamous cell carcinoma, endometrial cancer, and cervical cancers.

Emerging evidence suggests that BCL6B is closely associated with the initiation, progression, and clinical outcomes of multiple malignancies, including hepatocellular carcinoma (HCC), gastric cancer (GC), breast cancer (BC), colorectal cancer (CRC), lung cancer (LC), thyroid cancer (TC), cervical cancer (CC), renal cell carcinoma (RCC), differentiated thyroid carcinoma (DTC), and lymphomas. In many of these cancers, BCL6B expression is silenced through promoter CpG island hypermethylation, a common epigenetic mechanism that contributes to tumor suppressor gene inactivation. Xiang T et al. 9 identified BCL6B as a frequent target of DNA methylation. Including CRC, HCC, GC, BC, CC, LC, and Xuanwei lung cancer. With the exception of differentiated thyroid carcinoma (DTC), where BCL6B has been reported to be upregulated, studies in other investigated cancer types consistently demonstrate downregulation of BCL6B expression. This discrepancy may be attributed to the endocrine-related nature of DTC, suggesting that BCL6B expression may be modulated by hormonal or tissue-specific regulatory mechanisms unique to endocrine malignancies.

### Research on BCL6B in Hematologic Malignancies

As noted earlier, BCL6B contains all three essential domains required for sequence-specific DNA binding and transcriptional repression, suggesting that it may share target genes and regulatory functions with BCL6. Given their structural and functional similarities, it has been hypothesized that BCL6B may also play a role in hematological malignancies, particularly lymphomas 15.

In 2002, Sakashita C et al. 1 reported that BCL6B mRNA is expressed in specific immature B cell lines and erythroleukemia cell lines, implying a potential role in hematopoietic lineage differentiation and transformation. These findings raise the possibility that alterations in BCL6B expression or function may be linked to chromosomal abnormalities involving 17p13.1 in leukemia or other hematologic cancers. Nevertheless, direct evidence of BCL6B's involvement in hematologic tumorigenesis remains limited, warranting further mechanistic and clinical investigations.

### Research on BCL6B in Gastric Cancer (GC)

In 2012, Xu L et al. 16 identified BCL6B as a novel methylated gene in human cancers using methylation-sensitive representational difference analysis (MS-RDA). The results showed that BCL6B is silenced or downregulated in all nine GC cell lines examined, while it is highly expressed in normal gastric tissues—a difference attributed primarily to promoter hypermethylation. Functional studies demonstrated that restoring BCL6B expression in GC cell lines inhibited colony formation and viability, induced apoptosis, and significantly suppressed tumorigenesis in nude mice, confirming **its role as a** tumor-suppressive role in GC.

Additionally, multivariate COX regression analysis indicated that BCL6B methylation serves as an independent prognostic **factor** associated with poor survival in GC patients. Expanding on this work, Yang Q et al. 17 detected BCL6B promoter methylation in the plasma DNA of GC patients, suggesting its potential as a non-invasive biomarker. However, univariate analysis found no significant correlation between BCL6B promoter methylation status and clinicopathological features or survival outcomes, highlighting the need for further validation.

In contrast, Deng J et al. 18 observed a strong association between BCL6B promoter methylation and reduced survival in GC patients. To investigate upstream regulatory mechanisms, researchers from Fujian Medical University showed that TET1-mediated demethylation restores BCL6B expression, thereby suppressing GC progression. BCL6B expression also appeared to modulate GC cell sensitivity to chemotherapeutic drugs 19.

In an *in vivo* study, Cai WY 20 demonstrated that promoter methylation downregulates BCL6B, activating chronic inflammation and promoting benzo[a] pyrene-induced gastric carcinogenesis in mice. Treatment with 5-Aza-2'-deoxycytidine (5-Aza) reversed this methylation and reactivated BCL6B expression, exerting tumor-suppressive effects. However, the high toxicity of 5-Aza necessitates careful clinical consideration.

### Research on BCL6B in Hepatocellular Carcinoma (HCC)

In 2014, Jia W et al. 21 reported that BCL6B is abundantly expressed in normal liver tissue but downregulated in six of nine hepatocellular carcinoma (HCC) cell lines, again correlating with promoter hypermethylation. Ectopic expression of BCL6B significantly suppresses HCC cell proliferation, invasion, metastasis, and angiogenesis, and reduced lung metastases in orthotopic mouse models. These findings underscore its critical role in suppressing liver cancer progression.

Mechanistically, BCL6B promotes apoptosis via activation of caspase cascades and cleavage of poly (ADP-ribose) polymerase (PARP), and inhibits metastasis by upregulating tumor suppressors such as E-cadherin, OB-cadherin, HTATIP2, and TRPM1, while downregulating VEGFA. Importantly, BCL6B expression is negatively associated with advanced tumor stage, metastasis, and poor prognosis in HCC patients. In 2015, Weilin W et al. 22 investigated the physiological role of BCL6B in liver fibrosis, finding that its expression is significantly reduced in HCC tissues and rat fibrosis models. BCL6B restoration alleviated liver inflammation, reduced fibrotic markers, modulated hepatocyte growth factor levels, and suppressed hematopoietic stem cell activation—further supporting its diagnostic and therapeutic potential. Xin L et al. 23 provided additional evidence of epigenetic regulation of BCL6B in HCC. They showed that BCL6B suppresses cell proliferation, induces G1/S cell cycle arrest, and promotes apoptosis. Transcriptomic profiling revealed that BCL6B upregulates EGR1, which activates the p53 signaling pathway, thereby contributing to apoptosis induction. Notably, BCL6B enhances HCC cell sensitivity to 5-fluorouracil (5-FU). In clinical retrospective studies, BCL6B methylation was identified as an independent risk factor for post-thermal ablation metastasis and was strongly associated with poor prognosis in HCC patients. These studies collectively establish BCL6B as a tumor suppressor and prognostic biomarker in HCC, with potential therapeutic applications via epigenetic reactivation strategies.

### Research on BCL6B in Colorectal Cancer (CRC)

Early studies on BCL6B in colorectal cancer (CRC) revealed that its promoter methylation leads to transcriptional silencing, thereby promoting CRC progression. Re-expression of BCL6B suppresses CRC cell proliferation, invasion, and migration by activating the p53 signaling pathway, inducing G1/S phase arrest 24. Clinical data also suggest that BCL6B methylation correlates with advanced tumor stage, lymph node metastasis, and 5-fluorouracil (5-FU) resistance, indicating its value as a prognostic and predictive biomarker for chemotherapy response. Subsequent studies 25-27 further explored the link between BCL6B methylation and clinicopathological features in CRC patients 28, 29. Research teams from Chongqing Medical University provided deeper mechanistic insights. They demonstrated that BCL6B suppresses CRC progression by inhibiting the PI3K/AKT signaling pathway, upregulating E-cadherin, and downregulating Cyclin D1 and MMP-9. These changes enhance cell-cell adhesion and reduce metastatic potential. Additionally, BCL6B may modulate the Wnt/β-catenin pathway, further attenuating CRC cell proliferation and migration.

### Research on BCL6B in Breast Cancer (BC)

In 2017, Anne BK et al. 30 identified BCL6B as a gene with metastasis-specific mutations through genomic profiling of breast cancer (BC) progression. Follow-up studies in 2018 revealed that BCL6B functions as a suppressor of breast cancer metastasis, likely via upregulation of E-cadherin and downregulation of VEGFA. In 2020, Jin H et al. 31 reported that ectopic expression of ZBTB16 can form heterodimers with BCL6B, enhancing its tumor-suppressive effects and antagonizing BCL6-mediated oncogenic functions.

In 2022, Li L et al. 32 demonstrated that BCL6B expression is reduced in breast cancer due to CpG island methylation. Functionally, BCL6B inhibits halting proliferation, inducing apoptosis, and inhibiting EMT and stemness. Intriguingly, BCL6B modulates the type I IFN receptor (IFNAR), activating downstream interferon-stimulated genes (ISGs) and promoting macrophage polarization. Mechanistic insights suggest that BCL6B suppresses CD24 and CD47 expression, enhancing macrophage phagocytic activity by increasing antibody binding sensitivity, revealing a novel link between immune checkpoint modulation and BCL6B function in breast cancer.

### Research on BCL6B in Other Solid Tumors

Research on BCL6B in other solid tumors remains in the exploratory phase, with limited studies available. Juan W et al. 33 identified BCL6B hypermethylation in Xuanwei LC, suggesting it as a diagnostic biomarker. In 2021, Li L et al. 34 found that BCL6B inhibits proliferation, migration, invasion, and promotes autophagy in CC by regulating FIP200 and Bcl-XL. BCL6B also increases sensitivity to paclitaxel, cisplatin, and 5-FU. In 2022, Xin T et al. 35 dreported that BCL6B silencing due to promoter methylation inhibits apoptosis and promotes tumor progression. BCL6B downregulates stemness-related genes such as NANOG, KLF4, CD44, and ABCG2. In 2023, Lantao Zh et al. 36 showed that BCL6B downregulation in LC cells promotes proliferation and invasion. Mechanistically, circTFF1 sponges miR-29c-3p, upregulating DNMT3A to methylate BCL6B and enhance tumorigenesis. In the same year, Zijun Zh et al. 37 found that BCL6B is overexpressed in DTC compared to normal tissues, with elevated expression associated with poor prognosis. Notably, BCL6B negatively correlates with CD8+ T cell infiltration, suggesting a unique immune modulation role in DTC, potentially related to the endocrine nature of thyroid cancer.

## Research Progress on BCL6B in Immune Responses

The BCL6 gene encodes a transcriptional repressor protein that plays a critical role in regulating the expression of genes in lymphocyte and myeloid lineages 38-40. Alterations in the BCL6 gene are strongly linked to diffuse large B-cell lymphoma (DLBCL), where it functions as a recognized oncogene 39, 41. *In vivo*, BCL6 is essential for germinal center formation, and it modulates immune responses by regulating the differentiation of B cells, T cells, and bone marrow cells. Importantly, BCL6 acts as a key suppressor of Th2 responses and inflammatory processes 42-44.

As the closest homolog to BCL6, BCL6B is believed to exert important immunomodulatory functions. BCL6B mRNA is detected in naive CD4^+^and CD8^+^T cells 7 as well as in early B-cell lines and activated lymphocytes 1, 6. Functional studies suggest that BCL6B participates in naive CD4^+^T cell activation [7]and is critical for the immune function of CD8^+^T cells 45. Notably, *BCL6B*-deficient mice exhibit CD8⁺ T-cell exhaustion, highlighting its indispensable role in maintaining cytotoxic T cell function 46.

In addition, BCL6B is implicated in the regulation of macrophage and hematopoietic progenitor responses to IL-6, potentially via mechanisms shared with BCL6, including suppression of IL-6 synthesis 38. These findings collectively point to a broad regulatory role of BCL6B across both adaptive and innate immunity.

BCL6B belongs to the BTB-ZF (Broad-Complex, Tramtrack, Bric-à-brac - Zinc Finger) protein family. Members of this family are recognized as critical transcriptional regulators of hematopoietic development and differentiation 47. They guide lineage commitment of multipotent progenitors into B cells, T cells, and their subsequent differentiation into CD4⁺ and CD8⁺ effector subsets, thereby shaping primary immune responses.

BCL6B expression is also conserved in non-mammalian species, indicating an evolutionarily conserved role in immune regulation. In grass carp, BCL6B expression has been detected in immune-related organs including the gills, intestine, spleen, and head kidney, suggesting a systemic immunological role. Furthermore, several splice variants of BCL6B (BCL6BX1, BCL6BX2, and BCL6BX3) have been identified, with expression levels significantly upregulated in response to polyinosinic acid (PolyI) and lipopolysaccharide (LPS) challenge in the spleen 48. These findings suggest that BCL6B and its isoforms contribute to innate immune defense mechanisms in teleosts, providing a model for exploring its conserved functions.

### Research on BCL6B in T Cell Activation and Differentiation

BCL6B regulates the expression of multiple genes in T cells, thereby influencing their proliferation, differentiation, and functionality. For instance, BCL6B suppresses key genes within the TCR signaling pathway, modulating T cell activation and proliferation. Additionally, BCL6B affects T cell effector function by regulating cytokine gene expression 7.

#### Role of BCL6B in CD8^+^ T Cells

In 2005, Peter M et al. 45 reported that BCL6B enhances secondary immune responses by promoting CD8⁺ memory T cell proliferation and increasing effector cell numbers. Mice lacking BCL6B exhibit impaired CD8^+^ T cell memory responses. In both human and murine memory CD8⁺ T cells, BCL6B mRNA is expressed at lower levels than HPRT mRNA, suggesting a role in suppressing IL-2-mediated signaling.

While BCL6B-deficient mice show normal primary CD8⁺ T cell responses to viral infection, their memory recall responses are markedly impaired in both *in vivo* and *in vitro* settings 46. These mice also display abnormal CD8⁺ T cell differentiation and reduced granulocyte-macrophage (CFU-GM), erythroid (BFU-E), and multipotent progenitor cells (CFU-GEMM) in the bone marrow. Conversely, increased hematopoietic progenitor cell (HPC) numbers and proliferation are observed in the spleen, suggesting that BCL6B indirectly regulates HPC homeostasis via CD8⁺ T cells 46. BCL6B-mediated self-renewal of CD8⁺ T cells likely contributes to the expansion of effector precursors in secondary lymphoid organs, facilitating robust immune responses 7. Additionally, BCL6B-deficient mice exhibit abnormal proliferation of bone marrow progenitor cells, further supporting the intrinsic dependence of CD8⁺ T cells on BCL6B. 46 Recent studies have also revealed a negative correlation between BCL6B expression and surface molecules on CD8⁺ T cells, though no clear association was found with cytotoxic effector molecules. These findings imply that BCL6B may regulate CD8⁺ T cell activity by modulating their surface phenotype rather than directly affecting cytotoxicity 37.

#### Role of BCL6B in *BCL6B* in CD4^+^ T Cells

In 2004, Mikio T et al. 7 generated BCL6B-deficient mice (BCL6B-KO) and transgenic mice expressing BCL6B-cDNA under the control of the lymphocyte-specific protein tyrosine kinase (LCK) proximal promoter (LCK-BCL6B) to explore its role in CD4⁺ T cell function. Their results revealed that BCL6B is essential for optimal TCR-induced proliferation of naive CD4⁺ T cells *in vitro*, but it is dispensable for memory CD4⁺ T cells. BCL6B may also counteract BCL6-mediated repression of antigen-driven naive CD4⁺ T cell activation by forming BCL6/BCL6B heterodimers.

Moreover, ectopic expression of BCL6B leads to hyperproliferation of naive CD4⁺ T cells following TCR engagement, whereas memory CD4⁺ T cells remain unaffected. These findings underscore BCL6B's critical role in naive CD4⁺ T cell activation and expansion, reinforcing its functional distinction from BCL6.

### Research on BCL6B in B Cell Activation and Differentiation

B cell development from hematopoietic stem cells (HSCs) to memory cells or plasma cells is tightly orchestrated by a network of transcription factors. For example, PU.1 (Purine-rich box 1) is tightly orchestrated by a network of transcription factors 49. In the bone marrow, differentiation of HSCs into common lymphoid progenitors is regulated by IKZF1 and SPI1^50^. While E2A 50 and early B-cell factor (EBF) 51 are essential for the transition of pre-B cells to immature B cells, while paired box protein 5 (PAX5) is a critical transcription factor regulating this process 52.

Given its classification as a BTB/POZ-ZF transcription factor, BCL6B may influence B cell development and activation. In 1993, Ye et al. 3 identified BCL6B as a potential proto-oncogene implicated in the pathogenesis of non-Hodgkin's lymphoma in B cells, indicating its relevance in B cell biology. Additionally, BCL6B may function via the Notch signaling pathway, which plays a vital role in T/B cell lineage specification. Inactivation of Notch1 in mice results in reduced thymic T cell output and ectopic B cell expansion in the spleen, although bone marrow B cell development remains unaffected 53. This suggests that BCL6B may regulate early B cell lineage commitment by modulating Notch pathway activity.

Members of the POK/ZBTB family, including BCL6B, can form homo- or heterodimers via their BTB/POZ domains 12, 54. Experimental studies suggest that the BTB/POZ domain alone is sufficient to mediate POK (POZ and Krüppel) protein self-association 4. These dimeric interactions are vital for DNA binding, subcellular localization, and transcriptional repression. For instance, BCL6B/BCL6 heterodimers are thought to suppress expression of cell cycle genes in germinal center B cells, facilitating continued proliferation and antibody production during immune responses. Moreover, these proteins may inhibit STAT-dependent transcription by binding to the Gamma-Activated Sequence (GAS) motif, which regulates genes involved in immunity, apoptosis, and antiviral defense 13.

*BCL6B* expression has been detected in early-stage B cell lines (e.g., MTD5) but is absent in mature B cell lines 1, suggesting its role is restricted to early B cell development. Furthermore, BCL6B may promote megakaryocyte differentiation, potentially through regulation of bone marrow progenitor cell fate.

### Research on BCL6B in Macrophages (Mφ)

In 2022, Li L et al. 32 discovered that CpG methylation suppresses BCL6B expression in breast cancer using database analysis. BCL6B exhibits antitumor properties, including inhibition of proliferation, cell cycle arrest, induction of apoptosis, and suppression of epithelial-mesenchymal transition (EMT) and cancer stemness. Importantly, BCL6B also plays a role in innate immunity. It activates the type I interferon receptor (IFNAR) pathway, inducing the expression of interferon-stimulated genes (ISGs) and promoting macrophage polarization. Mechanistically, BCL6B enhances macrophage phagocytosis by downregulating the immune checkpoint molecules CD24 and CD47, thereby improving the efficacy of antibody-based therapies. These findings highlight the involvement of BCL6B in macrophage-mediated antitumor immunity and its interaction with immune checkpoint regulation.

## Research Progress on BCL6B in the Renewal of Spermatogonial Stem Cells (SSCs)

In 2006, Oatley JM et al. 55 identified BCL6B as a critical regulator of SSC self-renewal and survival, with its loss leading to spermatogenesis defects *in vivo*. These findings suggest that BCL6B may regulate functions in other stem cells and undifferentiated cell populations, acting as a conserved regulator of SSC self-renewal in mammals. Notably, long-term culture systems for SSC self-renewal have so far proven effective only in mice, rats, and hamsters 56 .In rats, BCL6B, like the transcription factor ETV5, is regulated by glial cell line-derived neurotrophic factor (GDNF), influencing SSC maintenance and survival 57 .In mice, GDNF upregulates BCL6B transcription through Src family kinase (SFK) signaling 58, while fibroblast growth factor 2 (FGF2) promotes SSC self-renewal by activating both ETV5 and BCL6B through MAP2K1 signaling in a GDNF-independent manner 59. Further studies showed that fibroblast growth factor 9 (FGF9) acts as a critical regulator of SSC proliferation by upregulating ETV5 and BCL6B expression through P38 MAPK phosphorylation 60. Additionally, BCL6B inhibits the transcriptional activity of ETV2 by binding to its promoter region 61, 62. Studies have also shown that in mice, ROS sustain SSC self-renewal by generating a positive feedback loop through the MAPK14/MAPK7/*BCL6B* pathway. Furthermore, NADPH oxidase 1 (NOX1), a key effector of BCL6B 63, produces ROS, which play a more critical role in SSCs than mitochondria-derived ROS 64. In goats, studies found that the combined supplementation of GDNF, insulin-like growth factor 1 (IGF1), and base fibroblast growth factor (bFGF) enhances *in vitro* SSC culture while inhibiting uncontrolled somatic cell proliferation 65. Additionally, the RAS/ERK1/2 pathway is crucial for maintaining dairy goat SSC self-renewal by regulating ETV5 and BCL6B 62. These findings underscore the critical therapeutic potential of BCL6B in addressing reproductive system disorders.

## Research Progress on BCL6B in Angiogenesis

The high expression of BCL6B in vascular endothelial cells suggests a potential role in angiogenesis. In 2012, BCL6B was identified as a mediator linking VEGF and Notch signaling pathways. Database analyses have shown that BCL6B is highly expressed at the mRNA level in human endothelial cells. Studies suggest that *BCL6B* is induced by VEGF-A, binds to the Notch signaling factor CBF1, and promotes CBF1 degradation via ubiquitination by the CBF1-CUL3 complex, thereby downregulating Notch signaling and promoting angiogenesis 11. Subsequent research demonstrated that heat shock protein 90 beta (HSP90β) positively regulates angiogenesis by stabilizing angiogenic gene mRNAs, including* BCL6B*, in a protein kinase D2 (PRKD2)-dependent manner 66. A 2017 study found that BCL6B is upregulated by hypoperfusion and non-perfusion during the acute ischemic phase, exerting protective effects in a rat model 67. Research on ocular vascular diseases indicates that BCL6B regulates retinal swelling and choroidal neovascularization by modulating VEGF-Notch signaling 53. While the exact role of BCL6B in endothelial cells remains unclear, evidence suggests that it facilitates angiogenesis and differentiation by modulating key regulatory factors. For example, BCL6B suppresses the transcriptional activity of ETV2, a key transcription factor for endothelial lineage development, by binding to its promoter region, thereby hindering the differentiation of human-induced pluripotent stem cells (hiPSCs) and inhibiting endothelial cell (EC) differentiation and vessel organoid (VO) development 61. While the exact mechanisms remain unclear, current evidence suggests that BCL6B promotes angiogenesis and endothelial differentiation by modulating critical regulatory pathways.

## Conclusion

The BCL6B gene, a member of the ZBTB (zinc finger and BTB domain-containing) protein family, functions as a multifunctional transcription factor with significant implications in both fundamental and clinical research. As a homolog of BCL6, it represses gene expression through its BTB/POZ domain, central region, and zinc finger domain. BCL6B modulates key signaling pathways, including Notch, VEGFR, STAT, PI3K/AKT, and P53, that play critical roles in regulating cellular proliferation, differentiation, apoptosis, and tumorigenesis. These characteristics make BCL6B an essential regulator in gene expression and a promising target for therapeutic intervention.

In tumor biology, BCL6B has demonstrated notable tumor-suppressive activity. Aberrant expression of BCL6B, frequently caused by promoter hypermethylation, is reported in several solid tumors, such as hepatocellular, gastric, breast, and colorectal cancers, as well as hematological malignancies. These epigenetic alterations lead to reduced expression of BCL6B, correlating with increased tumor proliferation, migration, and invasiveness. Reactivating BCL6B expression or reversing its promoter methylation has been shown to reduce tumor aggressiveness, underscoring its therapeutic potential. Moreover, reduced BCL6B levels in cancers like liver and gastric cancer are strongly linked to poor survival outcomes, indicating its potential as a diagnostic and prognostic biomarker (Table 1).

Beyond oncology, BCL6B plays a pivotal role in immune regulation, particularly in the T and B lymphocytes. Research suggests that BCL6B enhances secondary immune responses by regulating CD8^+^ T cell memory and self-renewal, while also influencing CD4^+^ T cell proliferation and antigen-driven activation. These findings suggest that BCL6B may contribute to autoimmune disease pathogenesis, while also serving as a promising target for immunotherapeutic strategies aimed at enhancing anti-tumor immunity (Table 2).

In angiogenesis, BCL6B has emerged as a key regulatory factor. Its high expression in vascular endothelial cells and its regulation of VEGF and Notch signaling position it as a critical player in vascular biology. These functions suggest new therapeutic avenues for treating ischemic cardiovascular and cerebrovascular conditions. Furthermore, in regenerative medicine, contributes to the maintenance of stem cell homeostasis through signaling pathways such as GDNF and FGF highlights its therapeutic potential in addressing disorders involving stem cell dysfunction (Table 3).

**Figure 2:**
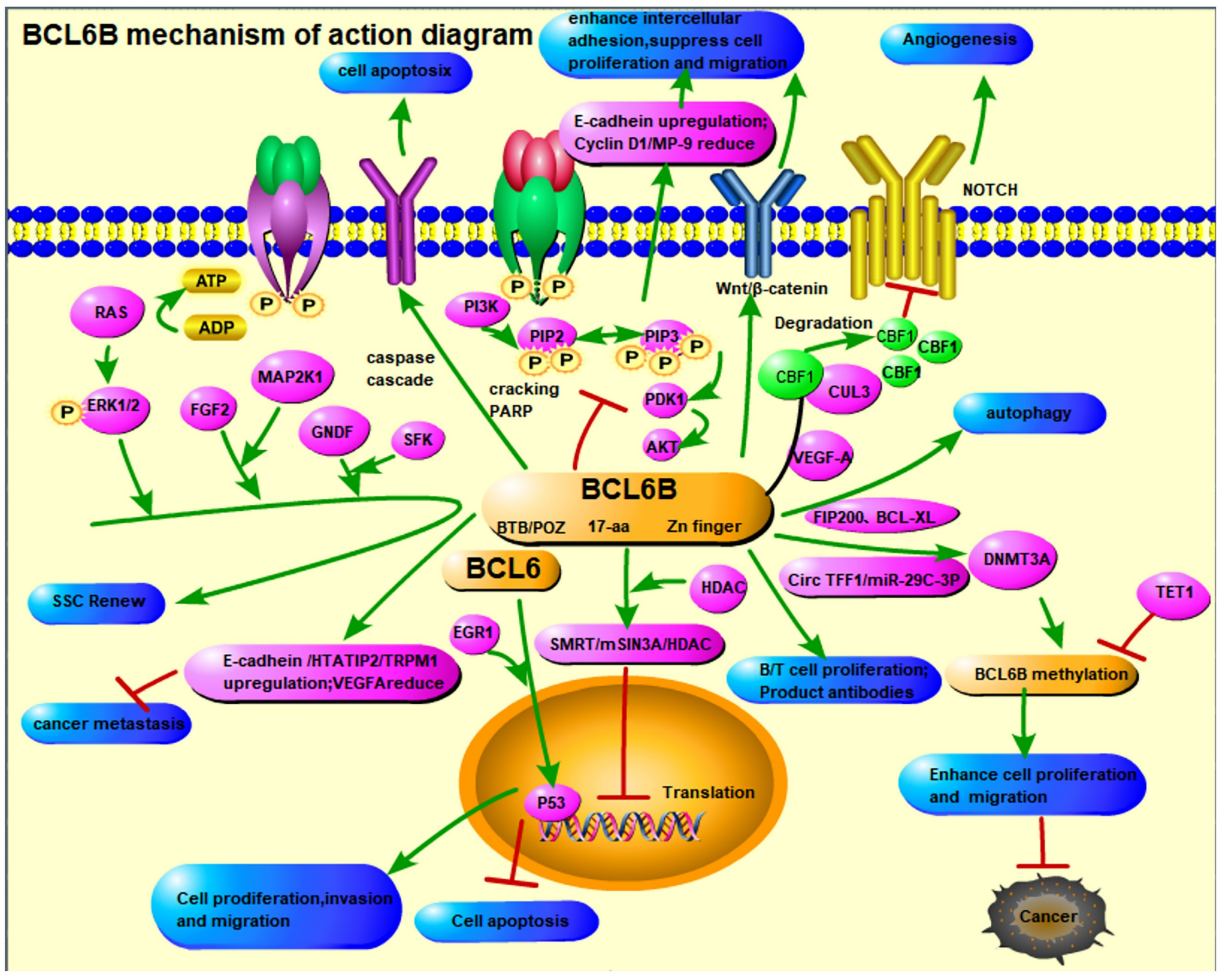
The Mechanism of Action Diagram of *BCL6B.*

Future research on BCL6B should expand mechanistic studies to elucidate its molecular role in tumorigenesis, immune modulation, and angiogenesis, with a particular focus on its epigenetic regulation and crosstalk among key signaling pathways (Figure 2). The use of advanced technologies, such as multi-omics approaches and single-cell sequencing, will provide a comprehensive understanding of BCL6B's temporal and spatial regulatory dynamics across different biological contexts. The development of cutting-edge models, including organoids and gene-edited animals, will be essential for validating BCL6B's roles in tumor suppression, immune regulation, and stem cell equilibrium. Such models will also accelerate drug discovery and inform the design of personalized therapeutic strategies.

Clinically, BCL6B holds considerable promise as a diagnostic biomarker and therapeutic target. Non-invasive diagnostic tools, such as liquid biopsies detecting BCL6B promoter methylation in circulating plasma DNA, could enable early cancer detection and prediction of treatment response. The development of targeted therapies, including small-molecule inhibitors and epigenetic modulators, offers a pathway to restore BCL6B function. Moreover, gene-editing technologies like CRISPR/Cas9 could be leveraged to precisely correct BCL6B mutations or aberrant methylation, further enhancing therapeutic precision.

Integrating interdisciplinary research will further expand the clinical utility of BCL6B. Its role in autoimmune diseases, angiogenesis, and regenerative medicine highlights its versatility as a therapeutic target. Combining immunotherapeutic approaches with BCL6B modulation could enhance anti-tumor immunity, while its regulation of vascular pathways offers innovative treatments for ischemic and vascular diseases. The integration of basic research with clinical applications will not only enrich our understanding of BCL6B but also pave the way for its translation into effective therapies, positioning BCL6B as a cornerstone of next-generation biomedical innovation.

As a multifunctional transcription factor, the BCL6B gene is of great significance in both basic and clinical research. Research on BCL6B, spanning tumor suppression, immune modulation, stem cell homeostasis, angiogenesis, molecular mechanisms, and therapeutic innovations, enhances our understanding of gene regulatory systems and support the development of innovative diagnostic and therapeutic strategies. Integrating basic research with clinical applications will further broaden the research prospects of BCL6B.

## Figures and Tables

**Table 1:** The role of BCL6B in solid tumors

Tumor Type	BCL6B Expression	Cellular Studies	Animal Studies	Epigenetic Modifications	Pathways/Factors	Clinical Applications	Reference
Gastric cancer (GC)	Reduced	Induces apoptosis, reduces migration	No direct evidence yet	Hypermethylation detected in plasma DNA	No direct evidence yet	Influence the sensitivity to chemotherapy drugs, reduction can amplify the inflammatory response and exacerbate gastric cancer induced by benzo[a]pyrene in the body.	17-19, 24
Hepatocellular carcinoma (HCC)	Reduced	Suppression of proliferation and invasion upon reactivation	Reduces tumor size in mouse models	Promoter hypermethylation linked to poor prognosis	Upregulate EGR1, activate p53	Increase sensitivity to 5-FU, improve inflammatory response during liver cell damage and liver injury, and reduce liver fibrosis.	21-23
Colorectal cancer (CRC)	Reduced	Inhibits cell cycle progression and migration	No direct evidence yet	CpG island methylation suppresses expression	Inhibit the PI3K/AKT pathway and the Wnt/β-catenin pathway.	Correlates positively with the late TNM stage and lymph node metastasis of patients' tumors; TNM stage, degree of differentiation, and the presence or absence of metastasis are all independent factors influencing the methylation rate of the *BCL6B* gene promoter region.	24, 26, 28, 29
Breast cancer (BC)	Reduced	Regulates macrophage activity, reduces EMT	Suppresses metastasis, improves immune response	Hypermethylation alters gene activity	Regulating IFNAR, stimulating interferon-stimulated genes (ISGs), downregulating CD24 and CD47, which favors the enhancement of macrophage phagocytic capacity.	No direct evidence yet	30-32
Cervical cancer (CC)	Reduced	Inhibit proliferation, growth, migration, invasion, and carcinogenicity, induce cell cycle arrest, and increase cell apoptosis, induce autophagy.	Suppress the growth of subcutaneous tumors and the formation of lung metastases.	Downregulated due to CpG methylation	No direct evidence yet	Enhanced the sensitivity of cervical cancer cells to paclitaxel, cisplatin, and 5-fluorouracil.	34
Lung cancer (LC)	Reduced	Inhibit cell proliferation, migration, and invasion.	No direct evidence yet	DNMT3A can promote the methylation of the BCL6B promoter.	CircTFF1 expression is upregulated and positively regulates the expression of DNMT3A through miR-29c-3p.	No direct evidence yet	36
Differentiated thyroid carcinoma (DTC)	Increased	Promote proliferation, apoptosis, migration	No direct evidence yet	No direct evidence yet	No direct evidence yet	No direct evidence yet	37

**Table 2:** The role of BCL6B in the immune system

Disease Type	BCL6B Expression	Cellular Studies	Animal Studies	Epigenetic Modifications	Pathways/Factors	Clinical Applications	Reference
Immune regulation	Altered regulation in T and B cells	Modulates immune cell differentiation	Reduces inflammation and autoimmunity in mice	No direct evidence yet	TCR signaling, cytokines	Immunotherapy target for autoimmune conditions	38, 45-53

**Table 3:** The role of BCL6B in other physiological contexts

Physiological Type	BCL6B Expression	Cellular Studies	Animal Studies	Epigenetic Modifications	Pathways/Factors	Clinical Applications	Reference
Spermatogonial stem cell renew	Increased	Increase SSC proliferation and impaired spermatogenesis	Enhances SSC expansion and spermatogenesis	No direct evidence yet	GDNF、p38 MAPK、RAS/ERK1/2	No direct evidence yet	55-62
Angiogenesis	High in vascular endothelial cells	Promotes endothelial cell proliferation	Enhances angiogenesis in mouse models	No direct evidence yet	VEGF, Notch	No direct evidence yet	11, 53, 61, 66
